# Neuromodulation and the Gut–Brain Axis: Therapeutic Mechanisms and Implications for Gastrointestinal and Neurological Disorders

**DOI:** 10.3390/pathophysiology31020019

**Published:** 2024-05-17

**Authors:** Baha’ Aljeradat, Danisha Kumar, Sulaiman Abdulmuizz, Mrinmoy Kundu, Yasser F. Almealawy, Dima Ratib Batarseh, Oday Atallah, Michelle Ennabe, Muath Alsarafandi, Albert Alan, Martin Weinand

**Affiliations:** 1 Global Neurosurgical Alliance, Tucson, AZ 85716, USA; bjaradat9@gmail.com (B.A.); danisha.kumar21@dmc.duhs.edu.pk (D.K.); muizzsulaiman@gmail.com (S.A.); mrinmoyk111@gmail.com (M.K.); almealawyyasser@gmail.com (Y.F.A.); dima_batarseh641999@outlook.com (D.R.B.); oday.atallah@yahoo.de (O.A.); mennabe@arizona.edu (M.E.); malsarafandi02@gmail.com (M.A.); 2 School of Medicine, The University of Jordan, Amman 11942, Jordan; 3 Dow Medical College, Dow University of Health Sciences, Karachi 74200, Pakistan; 4 College of Health Sciences, University of Ilorin, Ilorin 240003, Kwara, Nigeria; 5 Institute of Medical Sciences and SUM Hospital, Bhubaneswar 751029, India; 6 Faculty of Medicine, University of Kufa, Kufa P.O. Box 21, Iraq; 7 Department of Neurosurgery, Hannover Medical School, 30625 Hannover, Germany; 8 College of Medicine, The University of Arizona College of Medicine, Phoenix, AZ 85004, USA; 9 College of Medicine, Islamic University of Gaza, Rafa Refugee Camp, Rafa P.O. Box 108, Palestine; 10 Faculty of Medicine, Islamic University of Gaza, Gaza P.O. Box 108, Palestine; 11 Department of Neurosurgery, University of Arizona, Tucson, AZ 85724, USA; mweinand@arizona.edu; 12 College of Medicine, The University of Arizona College of Medicine, Tucson, AZ 85004, USA

**Keywords:** gut–brain axis, neuromodulation, deep brain stimulation, vagus nerve stimulation, irritable bowel syndrome, inflammatory bowel disease

## Abstract

The gut–brain axis (GBA) represents a complex, bidirectional communication network that intricately connects the gastrointestinal tract with the central nervous system (CNS). Understanding and intervening in this axis opens a pathway for therapeutic advancements for neurological and gastrointestinal diseases where the GBA has been proposed to play a role in the pathophysiology. In light of this, the current review assesses the effectiveness of neuromodulation techniques in treating neurological and gastrointestinal disorders by modulating the GBA, involving key elements such as gut microbiota, neurotrophic factors, and proinflammatory cytokines. Through a comprehensive literature review encompassing PubMed, Google Scholar, Web of Science, and the Cochrane Library, this research highlights the role played by the GBA in neurological and gastrointestinal diseases, in addition to the impact of neuromodulation on the management of these conditions which include both gastrointestinal (irritable bowel syndrome (IBS), inflammatory bowel disease (IBD), and gastroesophageal reflux disease (GERD)) and neurological disorders (Parkinson’s disease (PD), Alzheimer’s disease (AD), autism spectrum disorder (ASD), and neuropsychiatric disorders). Despite existing challenges, the ability of neuromodulation to adjust disrupted neural pathways, alleviate pain, and mitigate inflammation is significant in improving the quality of life for patients, thereby offering exciting prospects for future advancements in patient care.

## 1. Introduction

The gut–brain axis (GBA) is a bidirectional communication system that connects the enteric nervous system (ENS) of the gastrointestinal tract to the central nervous system (CNS) of the brain [1]. It affects a wide range of physiological processes in the neurological and gastrointestinal systems, such as digestion, metabolism, immune function, and even cognitive functions such as mood and behavior [2]. Understanding the intricate workings of the GBA is critical for investigating the mechanisms of various neurological and gastrointestinal disorders. Neuromodulation refers to a medical approach that precisely targets and modulates nerve activity within the nervous system to enhance neurological functioning and patient quality of life. This field is rapidly advancing, propelled by breakthroughs in neuroscience and medical engineering technology [3]. Different types of neuromodulation techniques are distinguished by their unique targets and methods of affecting the neurological system [4]. The aforementioned categories include deep brain stimulation (DBS), vagus nerve stimulation (VNS), Spinal Cord Stimulation (SCS), and Transcranial Magnetic Stimulation (TMS) [5]. DBS encompasses a surgical procedure wherein a device is implanted to administer targeted electrical impulses to distinct regions of the brain. DBS is primarily designed to address neurological conditions that primarily affect the brain, such as Parkinson’s disease and various movement problems [6]. VNS is a therapeutic approach that specifically focuses on the vagus nerve located inside the neck region. A VNS device is commonly employed in the management of treatment-resistant depression and epilepsy. It functions by delivering periodic and gentle electrical energy pulses to the brain through the vagus nerve [7]. SCS involves the utilization of a device that administers a low-level electric current to the spinal cord. The utilization of this treatment modality is commonly observed in cases of chronic pain syndromes, particularly those involving neuropathic pain [8]. TMS is an emerging modality of brain stimulation that entails the application of an electromagnetic coil in direct contact with the scalp. TMS is often employed within the field of psychiatry, with a particular focus on its application in the treatment of depression [9].

Additional types of neuromodulation include sacral nerve stimulation (SNS), a technique that transmits impulses to the sacral nerve to enhance bladder control [10]. Responsive nerve stimulation is a therapeutic approach that entails the continuous monitoring of neurological activity to administer personalized and immediate therapy [11]. Epidural motor cortex stimulation (EMCS) is mostly employed for pain management, whilst occipital nerve stimulation (ONS) is utilized to treat certain forms of headaches or migraines by targeting the occipital nerves [12,13]. Transcranial direct current stimulation (tDCS) and transcutaneous electrical nerve stimulation (TENS) are non-invasive methodologies developed to elicit brain and nerve stimulation, commonly employed in the domains of pain treatment and rehabilitation therapy [14,15]. Remote electrical neuromodulation (REN) is a nascent therapeutic modality that involves the remote administration of electrical pulses, typically employed to alleviate migraines or manage pain [16].

In this review, we explored the current literature on the GBA, neuromodulation, and the possible uses for neuromodulation for the management of GBA disorders covering indications, possible mechanisms of actions, outcomes, and adverse effects in an effort to put a spotlight on what we believe is a potentially promising field that could impact and improve the quality of life for millions of patients worldwide with diseases with few options for management, with many reports describing a newer understanding of the role the GBA plays in them.

## 2. Methodology

This article seeks to provide a comprehensive review of the use of neuromodulation for gastrointestinal and neurological diseases. Our search strategy involved searching through PubMed, Google Scholar, Web of Science, and Cochrane Library databases using different combinations of the following search terms (GBA, Neuromodulation, Deep Brain Stimulation (DBS), vagus nerve stimulation (VNS), sacral nerve modulation, Spinal Cord Stimulation (SCS), Transcranial Magnetic Stimulation (TMS), epidural motor cortex stimulation (EMCS), Occipital nerve stimulation (ONS), transcranial direct current stimulation (tDCS), transcutaneous direct current stimulation, remote electrical neuromodulation (REN), Gastrointestinal Diseases, Irritable Bowel Syndrome (IBS), Gastroparesis, Inflammatory Bowel Diseases, Neurodegenerative disease, Parkinson’s disease (PD), neuropsychiatric disorders, benefits, outcomes, mechanism of action, pathophysiology, functional GI disorders, microbiota). Results were then screened by titles and abstracts for relevance, and data were synthesized until a comprehensive review was produced that served to provide a sufficient summary of published articles, covering key points for each of the headlines, as agreed by the authors. The inclusion criteria included articles providing evidence and describing the role of various neuromodulatory techniques in the settings of neurological and gastrointestinal diseases to cover the effects of neuromodulation on the gut–brain axis, and articles that did not directly discuss the role of neuromodulation in such conditions, case reports, conference presentations, non-English literature, and letters to editors/comments were excluded. 

## 3. Discussion

### 3.1. The Gut–Brain-Axis

#### 3.1.1. Key Components of the Gut–Brain Axis

a. Enteric Nervous System (ENS):

The ENS constitutes an extensive network of intrinsic neurons distributed throughout the gastrointestinal tract [17]. It functions independently of the CNS and is responsible for regulating essential gastrointestinal processes [17]. These functions include peristalsis, nutrient absorption, and gut motility. The ENS acts as an autonomous control system for gastrointestinal functions [17].

b. Vagus Nerve:

The vagus nerve (cranial nerve X) serves as the primary neural pathway connecting the gut and the brain [18]. It consists of sensory and motor fibers that facilitate bidirectional communication [18]. Sensory fibers transmit information from the gut to the brain, relaying signals related to satiety, nutrient availability, and gastrointestinal discomfort [19]. Motor fibers convey instructions from the brain to modulate gut functions such as gastric secretion and motility [19]. This dual communication allows for continuous feedback and regulation between the gut and the brain.

c. Gut Microbiota:

The gut microbiota comprises a diverse community of microorganisms, including bacteria, viruses, fungi, and archaea, residing within the gastrointestinal tract [20]. This microbial population exceeds the number of human cells in the body [20]. 

The beneficial roles of gut microbiota are the facilitation of metabolism through metabolic pathways and enzymatic activity, the synthesis of vitamins and metabolites, and the inhibition of pathogens [21]. Dysbiosis and subsequent illness may occur due to an imbalance of the gut’s microbial flora, including the disruption of both harmful and beneficial bacteria, in addition to the loss of bacterial diversity [22]. Dysbiosis, through effects on certain cytokines like tumor necrosis factor, interleukins 6, and beta, can lead to an inflammatory process associated with an increase in the permeability of the intestinal wall and a depletion of tight junction (TJ) proteins [23,24]. Many animal and human studies shows that dysbiosis can also affect the onset and progression of several diseases, such as inflammatory bowel disease, autism, diabetes, and PD [25,26]. The gut microbiota actively participates in GBA communication by producing bioactive compounds and metabolites [27]. These include neurotransmitters, short-chain fatty acids, and other signaling molecules that influence neural activity, immune responses, and physiological processes within the host organism [27]. The gut microbiota’s role is integral to shaping both gastrointestinal and neurological functions. 

In humans, evidence of an interaction between gastrointestinal bacteria and brain cells was first shown more than 20 years ago in the observation that patients with hepatic encephalopathy often showed a dramatic improvement when they took oral antibiotics [28].

In the meantime, emerging data support the role of microbiota in influencing anxiety and depressive-like behaviors [29] and, more recently, dysbiosis in autism. In fact, according to the severity of the disease, certain changes in the microbiota are observed in patients with autism [30]. Data have been provided that both brain–gut and gut–brain dysfunctions occur, with the former being dominant, particularly in IBS [31].

**Figure 1:**
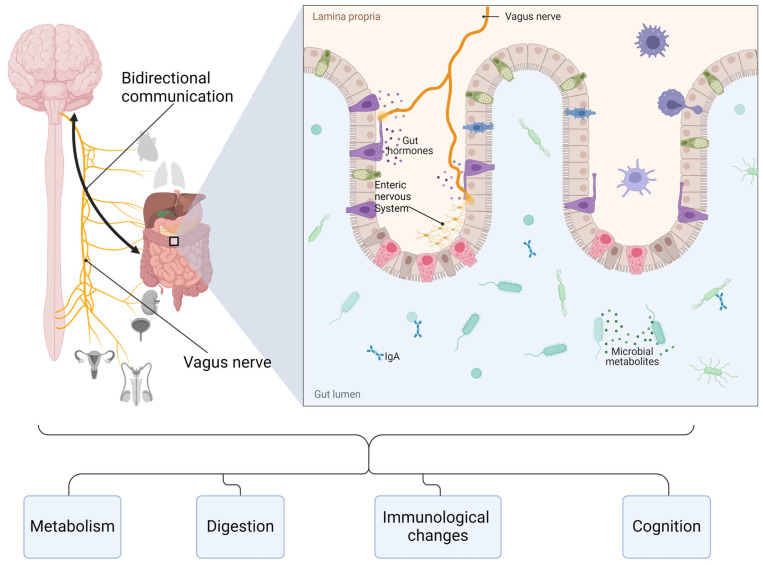
Key components and functions of the gut–brain axis. Created with BioRender.com (accessed on 14 May 2024).

Damage to the GBA can determine changes in intestinal motility and secretion, cause visceral hypersensitization, and lead to cell modification of the enteroendocrine and immune systems. Figure 1 shows key components and functions of the GBA. 

#### 3.1.2. Physiology of the Gut–Brain Axis

a. Neurotransmitters:

The GBA relies on a complex array of neurotransmitters to facilitate its communication pathways [32]. These neurotransmitters act as molecular messengers that transmit signals between the gut and the brain, thereby influencing various physiological processes. Key neurotransmitters involved in GBA communication include epinephrine, norepinephrine, dopamine, serotonin, and gamma-aminobutyric acid (GABA) [33]. Serotonin, primarily produced in the gut, is a critical player in regulating mood, cognition, and gastrointestinal functions [33]. GABA, an inhibitory neurotransmitter, can modulate stress responses and influence gut motility [33]. The precise balance of these neurotransmitters is essential for maintaining homeostasis within the GBA.

b. Hormones:

Hormones are integral components of the GBA, exerting profound effects on both gut and brain functions [34]. Cortisol, often referred to as the stress hormone, plays a central role in the GBA by regulating stress responses. Elevated cortisol levels can impact gut permeability and mucosal function [35]. Ghrelin, known as the hunger hormone, and leptin, which is responsible for appetite regulation and energy balance, are also featured prominently in GBA physiology. These hormones convey information about nutritional status to the brain, influencing food intake and energy expenditure [36]. The intricate interplay between these hormones within the GBA contributes significantly to overall homeostasis.

#### 3.1.3. Neural Communication between the Gut and the Brain

The neural communication pathways within the GBA constitute a remarkable system of information exchange that impacts various aspects of physiology and cognition. Understanding these intricate pathways is essential to grasp how gut-related signals influence brain function and vice versa.

a. Sensory Information Transmission:

Sensory information originating from the gut travels along dedicated neural routes, with the vagus nerve playing a central role. This cranial nerve serves as the primary conduit for transmitting visceral sensations and signals from the gut to the brain. As sensory input from the gastrointestinal tract ascends through the vagus nerve, it eventually reaches the brain stem [37]. Here, crucial processing occurs, enabling the relay of information to higher brain regions. The brainstem acts as a gateway for GBA signals, with connections to various brain areas that regulate autonomic functions, emotional responses, and homeostatic balance.

b. Brain Regions Influenced by GBA Signals:

Within the brain, sensory information from the gut can impact several key regions, including the following:

Hypothalamus: The hypothalamus serves as a central hub for regulating autonomic functions, hormonal responses, and appetite. GBA signals can influence hypothalamic circuits, modulating processes like satiety, thermoregulation, and stress responses [38].

Limbic System: The limbic system, particularly the amygdala and the hippocampus, plays a pivotal role in emotional and memory-related processes [39]. GBA signals can influence emotional states and memory consolidation, contributing to the intricate link between gut health and mood disorders.

Pre-Frontal Cortex: The pre-frontal cortex, responsible for executive functions and decision-making, is susceptible to GBA influence. Altered GBA signaling has been associated with cognitive impairments and behavioral changes [40].

#### 3.1.4. Pathophysiological Involvement of Brain–Gut Axis in Different Disorders

##### Irritable Bowel Syndrome (IBS)

Gut–brain axis disorders include common gastrointestinal (GI) disorders. IBS is one of these disorders and has been researched thoroughly. It presents as bowel function changes that can manifest as discomfort in the abdomen without detectable biochemical or structural changes [41]. The etiology of IBS is not fully understood, yet several factors are widely acknowledged to be linked to it. Unraveling the underlying mechanisms of IBS is crucial for crafting pharmacological treatments. Visceral hypersensitivity, gut–brain interplay, and microbiota dysbiosis all contribute to the pathogenesis of IBS [42]. 

Unfortunately, there is no definitive therapy for IBS. Typically, management involves reducing or avoiding exacerbating factors like specific medications, stress, and certain dietary patterns. However, GBA modulation is currently being explored as a promising avenue for novel treatment development [43]. 

Population studies have shown that IBS is very common [44,45]. For example, the Rome IV and III, IBS diagnostic questionnaires as well as 80 items of data were used in a recently conducted global study with 24 countries to assess indicators relating to GBA disorders. The findings show that 40% of the global population is affected by GBA disorders which affect individual quality of life and healthcare use [46]. In addition, the population of IBS is usually dominated by females in Western countries [47]. 

IBS includes changes in visceral hypersensitivity and GI motor abnormalities, which can cause various symptoms like diarrhea or constipation and abdominal pain [44]. Pathogenetic factors such as genetic susceptibility, GBA dysfunction, and innate immunity issues can also play a role in its pathogenesis. However, in many cases, it is difficult to determine which of these factors is contributing to the clinical picture and which one is not, especially given the differences between individual patients [48]. Therefore, for IBS, therapeutic approaches frequently address the patient’s primary or most bothersome symptom, rather than directly targeting the underlying pathophysiology, as seen in other organic gastrointestinal conditions [49].

Studies showed that disturbance of the structural and functional GBA can cause alterations in CNS reflexes and perceptual responses, which can potentially instigate GI disorders, such as IBS [50].

##### Inflammatory Bowel Disease (IBD)

Inflammatory bowel illness, which includes ulcerative colitis (UC), Crohn’s disease (CD), and microscopic colitis (MC), is alarmingly frequent, affecting roughly 1% of the global population and growing in prevalence [51]. IBD has a substantial influence on psychological well-being and social functioning as well. Both CD and UC involve significant inflammation and disruption of the gut immune system [52]. The vagus nerve, pelvic nerves, sympathetic innervation of the stomach, and intrinsic neurons of the ENS all have an impact on inflammation.

##### Gastroesophageal Reflux Disease (GERD)

Gastroesophageal reflux disease (GERD) is a common chronic disease wherein the regurgitation of stomach substances into the esophagus generates discomfort or obstructions. It is categorized into three forms: erosive esophagitis, non-erosive reflux disorder (NERD), and Barrett’s esophagus. Principal GERD pathophysiological mechanisms consist of (a) compromised esophageal motility (peristalsis) leading to esophageal clearance; (b) malfunction of the anti-reflux barricade at the esophagogastric junction (EGJ) caused by transient LES relaxations (tLESRs), hypotonic lower esophageal sphincter (LES), and/or discordance between LES and the crural diaphragm (e.g., the existence of hiatal hernia); and (c) gastric causes including postponed gastric emptying and gastric acid recess [53,54].

##### Parkinson’s Disease

Parkinson’s disease is a chronic disease involving the degeneration of dopaminergic neurons in the midbrain and the widespread accumulation of alpha-synuclein (α-syn) [55]. 

The gut microbiota was shown to have a role in affecting neurotrophic factors, controlling inflammation, and promoting the function of inflammatory cytokines, B cells, or T cells, thus contributing to myelination and microglial activation [56]. This is why it can affect behavior and cognition, increasing the risk of mental and neurological diseases [57].

Movement symptoms supporting the establishment of a PD diagnosis are usually preceded by other motor symptoms, in particular gastrointestinal symptoms, suggesting that the microbiome–intestine–brain axis contributes to the development of PD [58]. Synucleinopathy may start in the intestinal tissue and make its way to the brain with innervating autonomic fibers [59]. Disruptions to the normal balance of the microbial flora in the gut are believed to have a significant influence on the GBA. Recent studies suggest that gut-associated changes and processes can contribute to the development of Parkinson’s disease [60].

Alterations of the colon’s components have also been established to be related to Parkinson’s disease and other neurodegenerative diseases, according to a meta-analysis carried out by Gerhardt and colleagues [61]. 

A microbiota exhibiting a consistent composition characterized by elevated Bifidobacteria and Bacteroides levels, alongside diminished Firmicutes and Proteobacteria levels, is commonly linked with reduced lipopolysaccharide levels, indicating a healthy gut epithelium, despite large individual differences in the microbiota composition. However, there are often differences in the microbiota of patients with PD, which are similar to those observed in patients with inflammatory bowel disease [62].

##### Neuropsychiatric Disorders

Changes in the composition of human gut microbiota seem to be associated with neuropsychiatric and mood disorders [63] and neurotransmitter imbalances [64]. Additionally, it is reported that the gut microbiota maybe linked with symptoms of autism spectrum disorder (ASD); people with ASD are commonly affected by gut microbiota dysbiosis and gastrointestinal problems [65]. Moreover, individuals experiencing gastrointestinal discomfort are often prone to exhibiting mental comorbidities [63].

Studies show that there is an association between diet, nutrition, anxiety, and depression, suggesting that one possible preventative measurement or alternative treatment option addressing anxiety or depression can be through certain dietary changes. This is particularly true given the growing belief in the association between poor diet and the risk of developing mental illnesses [66]. Dietary preferences, perceptions of sweetness and fatty foods, as well as taste thresholds can also be influenced by stress and depression [67]. A longitudinal research study spanning 10 years in France revealed a correlation between inadequate nutrition and the onset of depression, showing that healthier dietary patterns are linked with fewer symptoms of depression [68]. To determine the direction of correlation, conflicting results have been seen in randomized control trials and prospective studies. According to a meta-analysis of prospective studies, consuming a high-quality diet, regardless of its specific form, featuring an increased intake of fish and vegetables, was associated with a lowered incidence of depression. Compliance with a healthy diet showed a dose-type association with this decreased risk. However, the meta-analysis showed that a lack of nutrition was not associated with increased depression risk, and significant differences were observed from one study to another [69].

Studies have also been performed to identify the relationship between gut microbiota and depression. The correlation between this mental disorder and the flora in the gut has been confirmed by Naseribafrouci et al., as they showed that patients who suffer major depressive disorder often exhibit elevated levels of the genera Oscillibacter and Alistipes [70]. 

##### Autism Spectrum Disorder (ASD)

Autism spectrum disorder represents a multifaceted neurodevelopmental condition defined by two main impairments: enduring challenges in social communication and interaction, alongside restricted and repetitive behavioral patterns [71].

Around 46–84% of ASD patients are reported to have gastrointestinal symptoms including diarrhea, constipation, and abdominal pain [72]. These symptoms are also reported to correlate with the severity of ASD [73,74], with dysbiosis being, at least partially, the cause of these symptoms [75], as it affects the microbiome’s ability to regulate certain metabolites that can affect both neurobiological conditions and gastrointestinal functions, like corticosterone, indolepyruvate, and ethylphenylsulfate [76,77]. 

Dysbiosis in early life possibly impacts early neurodevelopment significantly, potentially modifying brain–gut signaling and disrupting the integrity of the blood–brain barrier [78,79]. Additionally, in animal models, evidence suggests a bidirectional association wherein social behavior is affected by the gut microbiome, and social structures and interactions influence the microbiome’s functionality composition [80,81,82].

In one trial [83], and following Microbiota Transfer Therapy (MTT) for patients with ASD, an 80% decrease in gastrointestinal (GI) symptoms (abdominal pain, indigestion, diarrhea, and constipation) was observed. Notably, these improvements were sustained even 8 weeks post-treatment. Additionally, assessments of ASD behavioral symptoms exhibited substantial improvement post-treatment, with sustained enhancements observed at the 8-week mark. Furthermore, there was an increase in overall bacterial diversity post-MTT and an increased abundance of specific taxa such as Prevotella, Bifidobacterium, and Desulfovibrio. These changes were then noted to persist after 2 years of follow-up, with ASD symptom improvement being even more pronounced than the initial 8-week change [84]. 

### 3.2. Effects of Neuromodulation on Gut–Brain Axis

#### 3.2.1. Physiological Changes in the Gut–Brain Axis after Neuromodulation Including Changes in GI Physiology

**Figure 2:**
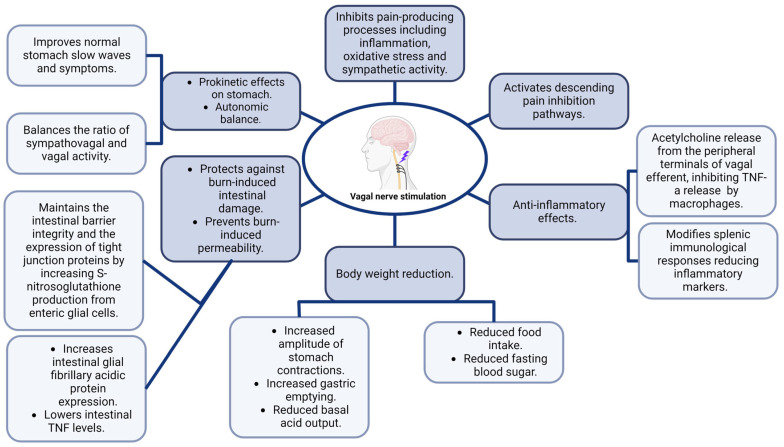
Summary of VNS effects on gut–brain-physiology. Created with BioRender.com (accessed on 14 May 2024).

**Figure 3:**
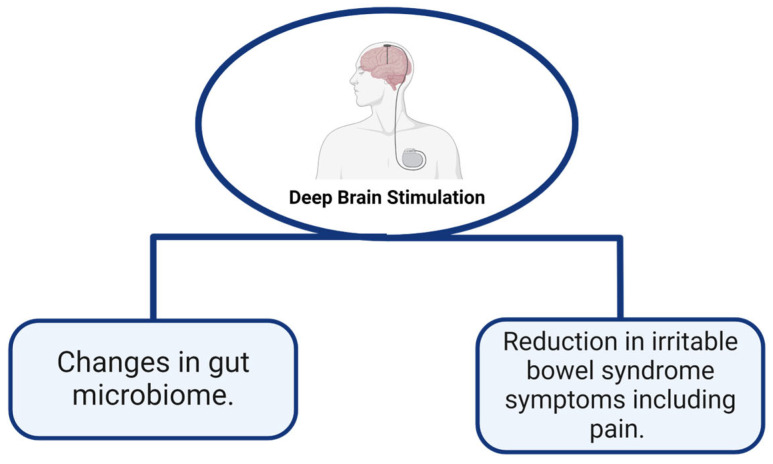
Effects of DBS on gut–brain axis. Created with BioRender.com.

Figure 2 and Figure 3 summarize key effects and changes in GBA physiology after neuromodulation. 

##### Neuromodulation Effect on Pain

In a case report, results from the assessment of a 55-year-old lady who received bilateral DBS in the anterior limb of the internal capsule and who had obsessive compulsive disorder and IBS are provided. Following brain stimulation therapy, there was a reported significant reduction in IBS symptoms. This relief was dependent on certain stimulation settings, showed consistency over time, and was not directly related to reductions in the symptoms of obsessive compulsive disorder. These findings imply that DBS has a positive effect on IBS [85].

The vagus nerve (VN) is increasingly being shown to affect nociceptive processing in the spinal cord and brain. It has been demonstrated that nociceptive mechanical and chemical stimuli cause vagal afferents to respond, which in turn causes the brainstem to reflect nociceptive signals [86,87]. Patients who receive VNS for epilepsy and depression usually report feeling less pain. Evidence suggests a connection between pain and VN activity since the VN is known to decrease pain-causing processes such as inflammation, oxidative stress, and sympathetic activity. Additionally, it stimulates parts of the brain that can block the “pain matrix” in the brain, which in turn modifies the way opioids function as an analgesic [88]. Nutrient content may influence the perception of painful visceral sensations in several gastrointestinal illnesses, including IBS, by enhancing the creation of aversive visceral memories via vagal afferent pathways [89].

VNS activates vagal afferents that go to the nucleus tractus solitarius (NTS), which suppresses experimentally produced pain. Descending pain inhibition pathways are then activated as a result of neurons in the NTS projecting to the Central Amygdala Nucleus (CAN), the Nucleus Raphe Magnus, and the locus coeruleus. Visceral pain is decreased by low-intensity VNS that particularly targets vagal afferent Ad fibers, indicating that a portion of the vagal afferents that innervate the viscera may have the ability to control visceral pain [90].

##### Effects of Neuromodulation on GI Motility and Permeability

It has been shown that it is possible to achieve prokinetic effects by increasing vagal tone by VNS, deep breathing, moderate-pressure massage treatment, or other exercises that have a substantial impact on heart rate and heart rate variability [91].

Four randomized sessions, comprising a control session, a conditioned stimulus (CS), a CS paired with transcutaneous auricular vagus nerve stimulation (taVNS), and a CS mixed with sham electrical stimulation (sham-ES), were carried out in research involving healthy individuals. Each session began with a period of fasting and ended with a test meal. While taVNS or sham-ES were given between 0 and 30 min after the meal, CS was delivered between 10 and 30 min after the meal. Assessing stomach slow waves and autonomic functioning involved recording the electrogastrogram and the electrocardiogram. The proportion of normal stomach slow waves and the symptom score were both considerably lowered by CS, and both were greatly improved by taVNS but not by sham-ES, according to the results. Additionally, CS raised the sympathovagal ratio and lowered vagal activity, while taVNS balanced these effects brought on by CS. These results imply that after exposure to a conditioned stimulus, taVNS has a prokinetic effect on stomach function and autonomic balance [92].

By stimulating enteric glial cells (EGCs), Costantini et al. [93] showed that VNS protects against burn-induced intestinal damage. Without altering systemic inflammation, this protection is achieved by controlling the reaction to damage inside the gut tissue itself, and the generation of cytokines in the spleen was shown to be not necessary for the maintenance of gut membrane integrity by VNS. Furthermore, S-nitrosoglutathione administration has been shown to produce outcomes that are comparable to those seen in animals that have received VNS, indicating that VNS may maintain the integrity of the intestinal barrier and the expression of proteins related to TJs by increasing the ability of activated EGCs to produce S-nitrosoglutathione [94].

Injuries that result in severe burns cause EGCs to become more activated, which increases the production of the mRNA for intestinal glial fibrillary acidic protein. When VNS is used alone, it increases intestinal glial fibrillary acidic protein expression and thus reduces burn-induced intestinal permeability and lessens gut histological damage. In one experiment, animals who had vagotomies before receiving VNS showed intestinal permeability levels similar to those of animals that had just had burns, demonstrating the protective effect of efferent vagal nerve transmission. Intestinal TJ proteins, myosin light chain kinase, and phosphorylated myosin light chain, all of which are essential for maintaining TJ integrity, as well as the elevation in intestinal TNF caused by burns, were likewise avoided and reduced by VNS. These results provide insight into the mechanism through which VNS prevents the loss of the intestinal barrier and subsequent intestinal inflammation [93].

Researchers found that VNS significantly lowers levels of intestinal TNF while successfully preventing trauma-induced intestinal permeability and intestinal damage in a mouse model modeling traumatic brain injury. Boosted levels of enteric glial fibrillary acidic protein also showed that VNS boosted the activity of EGCs in the same settings, which shows that the CNS can directly affect intestinal barrier failure [95].

Through the activation of myosin light chain kinase and the subsequent phosphorylation of myosin II light chain, which results in the contraction of the actin–myosin ring, inflammation can compromise the epithelial barrier and change the expression of TJ proteins. There is a lot of gut inflammation after burn injuries, which raises myosin light chain kinase and amplifies myosin II light chain phosphorylation, leading to the separation of TJ proteins. A protective effect of VNS is seen on the gut epithelial barrier with a “therapeutic window” for VNS intervention since it can be used before or within 90 min after burn damage. When used during this window, VNS can either stop barrier breakdown or promote its recovery. In these circumstances, the gut’s morphology, permeability, and protein expression are consistent. Similar to the sham-operated control group, in one study, VNS-treated mice showed decreased inflammation and localized TNF production in the gut [94].

##### Effects of Neuromodulation on GI Inflammation and Immunity 

Vagal efferents have been shown to have anti-inflammatory activity by Tracey’s team. In their study, they found that in a rat model of endotoxic shock, VNS caused the release of acetylcholine from the peripheral terminals of vagal efferents, which in turn inhibited the production of TNF-a by macrophages [96].

According to a study [97], low-frequency (5 Hz) VNS has been shown to treat colitis in rats. Additionally, VNS may have an anti-inflammatory impact on Crohn’s disease patients who have autonomic instability [98]. In one study, it was shown that in the majority (five out of seven) of patients with mild-to-moderate active Crohn’s disease, VNS resulted in clinical, biochemical, and endoscopic remission after 6 months. Additionally, VNS returned the autonomic balance to levels comparable to those in healthy people [99].

Endotoxin and intestinal inflammation have been proven to cause systemic inflammatory responses that can be reduced by VNS. Additionally, through interacting with the splenic sympathetic nerve, the vagus nerve indirectly modifies immunological responses in the spleen. Daily VNS for three hours spread over five days significantly decreased inflammatory markers and improved colitis symptoms in another rat study that involved colonic inflammation [19].

##### Effects of Neuromodulation on Gut Microbiota 

According to a systematic review, alterations in the relative abundances of the various bacterial species in the gut have been associated with the use of neuromodulation. The results also showed that neuromodulation therapies cause modest changes in the gut microbiome. However, they did not result in changes in the variety of species or the differences across microbial communities [100].

Dynamic changes in microbial composition are seen in studies examining the links between the gut microbiota and device-assisted treatments (DATs) for PD. Specific changes in gut bacterial composition result from the acute use of DATs such as DBS and Levodopa–Carbidopa Intestinal Gel (LCIG). Clostridium_XlVa and Parabacteroides are over-represented in DBS samples, possibly as a result of antibiotic usage. On the other hand, Pseudoflavonifractor is over-represented in LCIG treatment, whereas Escherichia/Shigella and Gemmiger are under-represented. With DBS, there is a rise in Euryarchaeota and Spirochaetes, whereas with LCIG, there is an over-representation of Prevotellaceae and Bacillus. The long-term usage of these treatments results in unique, diverse alterations. Notably, the microbiota’s reaction to DAT exposure varies depending on how long it continues. Although the exact causes of these alterations are not entirely known, they may be brought on by changing physiological responses and reciprocal interactions between gut microbiota and DATs [101].

In one study, electroacupuncture at certain acupoints, including DU20 and KI1, decreased pain and delirium-like symptoms in mice with a model of surgical pain and delirium brought on by a foot incision. Mice that experienced surgical pain and delirium-like behavior showed altered gut microbiota, spinal cord, somatosensory cortex, and hippocampus microglia activation and increased dendritic spine removal in the cortex. In addition to reducing pain and delirium-like symptoms, electroacupuncture therapy also balanced the gut microbiota, reduced microglia activation, and stopped dendritic spine removal. This shows that through regulating gut–brain connections and microglial activity, electroacupuncture may have therapeutic promise for treating surgical pain and associated delirium-like symptoms [102].

##### Effects of Neuromodulation on Weight Gain and Food Intake

According to a review article, multiple studies seem to agree that during vagal stimulation, body weight is significantly reduced [103]. On the other hand, one study showed no significant difference in food intake and weight gain after VNS stimulation in swine [104].

One study looked at how conscious rats’ long-term stomach motility, secretion, and weight management were affected by neuromodulation surgery employing a microchip (MC). Within two weeks after MC implantation, the use of MC-induced neuromodulation caused rats’ daily food intake to drop by 6% and their body weight increase to slow by 20%. Glucose levels in the fasting control group similarly dropped by 5.5%. When compared to the control group, the frequency of stomach contractions in the MC-treated rats remained consistent, but the amplitude of the contractions dramatically increased. In MC-treated rats, the maximum acid output (MAO) did not change, while the basal acid output (BAO) dropped by 29.25% without affecting the H+ concentration, and gastric emptying increased by 10% [105].

##### Special Consideration: Microbiota-Induced Vagus Nerve Stimulation in Cerebral Ischemia 

A potentially effective treatment for cerebral ischemia involves controlling microglia to reduce neuroinflammation. In one study, the signals of the GBA were examined regarding berberine-modulated microglia polarization after cerebral ischemia. The transient receptor potential vanilloid 1 (TRPV1) receptor, hydrogen sulfide (H2S) metabolism, and stimulation of the vagus nerve were all included in the study. A test using metabolomics was performed to investigate the brain microenvironment. The results showed that berberine restored behavioral impairments in rats with temporary middle cerebral artery blockage by modulating microglia polarization and reducing neuroinflammation via the microbiome. When given berberine, vagus nerve activity was increased, which could be inhibited by various antibiotic combinations, capsazepine, or sodium molybdate. VNS, accomplished by both assimilatory and dissimilatory sulfate reduction with enhanced synthetic enzymes, was linked to the synthesis of H2S generated by berberine. When berberine was supplied, the TRPV1 receptor’s sulfation in turn caused the vagus nerve to become activated and encouraged the production of c-fos and ChAT in the nucleus tractus solitarius. Additionally, sphingolipid metabolism was disturbed by the major metabolic change brought on by berberine in the cerebral cortex, hippocampus, and cerebral spinal fluid, which is a crucial feature that is altered by antibiotic therapy [106].

#### 3.2.2. Effects of Neuromodulation on the Gut–Brain Axis Disorders

Table 1 summarizes studies describing the main effects of neuromodulation on disorders in which the GBA has been suggested to play a role or demonstrate certain changes in association.

##### Irritable Bowel Syndrome (IBS)

Functional gastrointestinal disorders (FGIDs) are frequent and have a significant impact on a person’s quality of life. Although there are many approaches to treating distinct FGID symptoms, neuromodulation, a relatively recent treatment, has shown a favorable therapeutic impact on FGIDs.

The most common GI disorder, impacting approximately 7–21% of the population, is IBS [128]. Core pathophysiological mechanisms of IBS include visceral hypersensitivity (or modified pain perception), weakened brain–gut communication, changes in microbiota composition, and gastrointestinal motility [128]. Visceral hypersensitivity is considered the primary culprit behind abdominal pain or discomfort, believed to arise from heightened intestinal permeability and the activation of gut mucosal immunity. 

Tryptophan

Serotonin (5-hydroxytryptamine; 5-HT) acts as a prevalent transmitter in the gastrointestinal system, predominantly produced by enteroendocrine (EC) cells residing in the gut epithelium, accounting for 90–95% of the total 5-HT reservoir within the human organism. The synthesis of 5-HT is regulated by tryptophan hydroxylase (TPH) [129]. Moreover, the gut microbiota significantly influences 5-HT synthesis and release by modulating EC cells. In a rat model of post-infectious irritable bowel syndrome (IBS), quercetin demonstrated the ability to reduce the density of EC cells and downregulate TPH expression. Consequently, quercetin administration led to a decrease in 5-HT levels and the mitigation of visceral pain sensations experienced by IBS-afflicted rodents [130].

Transcutaneous auricular VNS (tVNS)

Forty-two individuals with constipation-dominant IBS were given taVNS [107]. The patients were randomly assigned to either undergo taVNS using silicon electrodes implanted at bilateral symba conchas or to undergo sham electrical stimulation (via the elbow). The application of taVNS resulted in an escalation in the weekly count of complete spontaneous bowel movements, the alleviation of abdominal discomfort, and enhancement in both overall IBS symptoms and quality of life. Furthermore, an improvement in the perception of rectal stimuli and the relaxation of the internal anal sphincter triggered by rectal distention was noted in the investigation, implying a vagal afferent and sacral efferent route. These effects were mediated through the vagal–sacral circuit in the following way: The NTS was stimulated, which then projected to other areas of the brain, which increased the activity of the sacral efferent, which acted on the rectum and anal sphincter [131]. A method involving the stimulation of the auricular vagal nerve (aVNS), using consistent parameters, was discovered to accelerate the movement of the lower part of the colon, which lacks direct innervation by the vagus nerve. This effect was accompanied by a simultaneous rise in the activity of neurons in the nucleus tractus solitarius (NTS) in a mouse model experiencing constipation induced by opioids [132]. These observations suggest that aVNS could impact the motility and sensation of the colorectum through both the vago-vagal and vago-sacral routes.

Tripolar Spinal Cord Stimulation

Coffin et al. have shown that the hyperexcitability of spinal nociceptive pathways causes visceral hypersensitivity in IBS patients [133]. The precise mechanism of action of SCS is unknown; however, based on animal research, one probable mechanism may be the suppression of pain pathways in the dorsal columns of the spinal cord [109]. Palecek has highlighted the significance of the dorsal column route in the control of visceral pain by selectively suppressing the response to a visceronoxious stimulus [108]. The precise method through which SCS modulates intestinal motility and causes pain alleviation is unknown. Auli et al. studied the impact of direct electric stimulation on enteric motor neurons in human intestinal strips in vitro [110]. They discovered that the activation of inhibitory neurons causes the release of nitric oxide and purine, whereas the stimulation of excitatory neurons causes the release of acetylcholine and tachykinins. Purine agonists and medicines that boost acetylcholine production and release, in comparison, have been demonstrated in tests to have significant analgesic effects [134].

##### Inflammatory Bowel Disease (IBD)

Vagal nerve stimulation possesses the capability to diminish inflammation within the intestinal tract through various mechanisms. Firstly, it initiates signals via afferent neurons, prompting the brain to engage efferent pathways, potentially involving sympathetic responses from the central nervous system [111]. Secondly, it directly activates efferent pathways of the vagus nerve. Thirdly, it triggers the release of neurotransmitters from the peripheral terminals of vagal afferents. Support for the influence of vagal efferents on enteric neurons stems from observations indicating that intestinal inflammation instigates a circuitry mediated by the vagus nerve, resulting in the activation of motor neurons associated with the inflamed region of the gut [135]. VNS decreased the mechanically induced inflammation of the small intestine in normal mice, mice with denervated spleens, and T-cell-deficient animals [112]. In Nicotinic Acetylcholine Receptor (7nAChR) knockout mice, VNS proved ineffective. Enteric neurons innervated 7nAChR-expressing macrophages, and 7nAChR activation lowered their excitability. According to the findings, resident macrophages are a target via which VNS mediates its anti-inflammatory activity.

##### Gastroesophageal Reflux Disease (GERD)

Transcutaneous electrical acustimulation (TEA)

Acute TEA at bilateral ST36 and bilateral PC6 acupoints has been shown to increase stomach accommodation and pace-making activity, as well as diminish post-prandial dyspepsia symptoms in GERD patients [136]. In patients with GERD, 4-week TEA at bilateral ST36 and bilateral PC6 alleviated reflux symptoms, increased distal esophageal motility, decreased the incidence of inefficient esophageal contractions during wet swallows, and enhanced stomach accommodation and pace-making activity [113]. The researchers concluded that the improvement in GERD symptoms was due to TEA’s integrative effects on various gastric processes, which were mediated through the vagal mechanism. In another study, TEA at ST36 and PC6 was combined with deep breathing training in GERD patients [114]. Four-week therapy with this combination technique reduced acid reflux and GERD symptoms while simultaneously increasing LES pressure and vagal activity and decreasing serum nitric oxide.

Transcutaneous abdominal electrical stimulation

Although the mechanism is unknown, it appears that transcutaneous abdominal electrical stimulation is intended to cause abdominal muscular contractions, hence increasing LES pressure. The pressure recorded via esophageal manometry of the lower esophageal sphincter (LES) encompasses the collective force exerted by both the LES and the crural diaphragm. It is speculated that certain stimulations may elevate the crural diaphragm pressure, yet empirical evidence supporting this hypothesis is lacking. In a preliminary open-label investigation, transcutaneous abdominal electrical stimulation demonstrated a noteworthy reduction exceeding 50% in acid exposure duration and DeMeester score among GERD patients who exhibited resistance to conventional proton pump inhibitor therapy [137].

##### Autism Spectrum Disorder

Autism spectrum disorder (ASD) is a neurodevelopmental disorder with a wide range of symptoms, including social deficits and confined, repetitive activities [115]. ASD therapy is notoriously difficult, and it may benefit from identifying underlying systems that overlap with those disrupted in other developmental disorders, which have more clear treatment choices. DBS in the prefrontal cortex, hypothalamic nucleus, and central thalamus has been proven in preclinical investigations to relieve VPA (valproate acid)-induced autism-like symptoms [138]. VNS is an FDA-approved therapy for reducing the severity of persistent epilepsy and depression, and it has emerged as a viable adjuvant therapy for people with autism [139]. ASD is usually associated with a dysregulated parasympathetic nervous system and decreased vagal tone, which is linked to autistic behavioral and linguistic impairments [140]. The use of VNS in children with epilepsy and ASD has yielded promising outcomes [141]. Several studies have shown that gamma-band response (30–80 Hz) is highly dependent on the cellular balance of excitation and inhibition (E/I) signal transduction [142], mediating several basic neural functions such as sensorimotor integration, perceptual integration, working memory, network synchronization, and higher-order cognition [143], all of which are disrupted in multiple ASD systems [144]. 

Vagus Nerve Stimulation

Seizure reduction was comparable amongst persons with and without autism in the biggest study of VNS treatment in individuals with ASD to date [141]. After 12 months of VNS therapy, 56% of people without autism had a 50% reduction in seizures, whereas 62% of those with autism had a 50% reduction in seizures. Individuals with and without autism had comparable gains in attentiveness, verbal communication, memory, and academic/professional success. Patients with autism, on the other hand, exhibited a considerably higher improvement in mood after 12 months of VNS therapy than persons without autism. The stimulation of the vagus nerve causes strong, phasic neuronal activity in the locus coeruleus, the principal source of norepinephrine in the CNS [145]. VNS increases norepinephrine levels in the hippocampus and cortex, which is consistent with VNS-dependent noradrenergic system activation [116]. Furthermore, VNS considerably raises levels of brain-derived neurotrophic factor (BDNF), a neurotrophin strongly associated with neural plasticity that is dysregulated in autistic people [146]. 

Transcranial Magnetic Stimulation (TMS)

In line with the impact of cortical development alterations in ASD, prefrontal and frontal cortex regions that play a role in social skills and language production undergo a spiking increase in plasticity and synaptogenesis between years 1 and 3 [117], which is typically when signs of autism associated with these functions emerge. TMS generates transitory, localized electrical fields in the cerebral cortex by electromagnetic induction, producing the depolarization and firing of local neurons [147]. Repetitive Transcranial Magnetic Stimulation (rTMS) generates numerous TMS patterns of pulses applied on a selected brain region at frequencies ranging from 0.5 to 20 Hz [148]. The long-term sustained suppression of excitability of the target cortex is produced through low-frequency rTMS, but at higher frequencies, rTMS causes the long-term stimulation of cortical excitability. rTMS could have clinical utility as an intervention in ASD. Some studies indicate that the low-frequency stimulation of the dorsolateral prefrontal cortex (DLPFC) can reduce repetitive behaviors, improve neurophysiological markers of perception, and reduce irritability; low-frequency supplementary motor area (SMA) stimulation can improve movement-related cortical potentials; and low-frequency stimulation of the premotor cortex can improve sensorimotor integration [118,119,120]. 

Transcranial electric stimulation (tES)

Transcranial electric stimulation modalities include tDCS, transcranial alternating current stimulation (tACS), and transcranial random noise stimulation (tRNS). tDCS employs a continuous mild electrical current to cause bidirectional, polarity-dependent changes in cortical areas, allowing for the measurement of effects at the cognitive, physiological, and motor levels [149,150]. tDCS can raise or reduce cortical excitability and/or specific brain oscillations. Gamma activity can also be modulated by tDCS. tRNS employs a low-intensity, biphasic current that is randomly alternating and applies it directly to the scalp with frequencies between 0.1 and 640 Hz [151]. While both tDCS and tRNS are successful in modifying cortical plasticity and excitability processes, tACS is uniquely associated with the frequency-specific modulation of oscillatory dynamics, demonstrated by its impact on gamma activity in both animal and human studies. It utilizes biphasic or sinusoidal currents at specific frequencies to synchronize with the brain’s intrinsic oscillatory activity, thereby entraining large neuronal populations [152]. tACS operates by aligning spiking activity with different stimulation frequencies, which coordinates neuronal firing with the applied electrical field, resulting in significant neuromodulatory impacts. Improvements induced by gamma tACS were closely linked to alterations in the blood oxygenation level-dependent (BOLD) activity within the stimulated M1 area [153]. Higher-order behavioral processes have also been targeted using tACS gamma-entrainment approaches. Hoy and colleagues discovered that after gamma-tACS, there is a selective improvement in the performance of working memory [154]. Since gamma activity is reduced in patients with ASD, utilizing gamma-tACS on these areas could potentially entrain and, to some extent, restore the activity of neurotypical gamma for ASD patients.

##### Parkinson’s Disease

Parkinson’s disease, which is a chronic and a progressive condition, is marked by the degeneration of numerous dopaminergic neurons within the circuitry of the basal ganglia. This reduction in dopamine levels leads to the manifestation of clinical motor symptoms, including tremors, bradykinesia, postural instability, stiffness, and impaired gait [155]. Rising clinical and epidemiological evidence suggests that “mild cognitive impairment” (MCI) may be present characteristically in the early stages of PD [156].

Deep Brain Stimulation

DBS is efficient in modulating abnormal basal ganglia motor circuit activity by acting on particular nuclei such as the globus pallidus interna, subthalamic nucleus, and the thalamus. This method entails implanting pacing devices that provide constant high-frequency stimulation of the targeted region. The STN, a critical motor relay component whose failure has been related to Parkinson’s disease symptoms, has been the most widely utilized target for DBS during the last decade [157]. Numerous studies have also demonstrated that STN DBS gives lasting symptom relief even 5 or 10 years following surgery, but with the worsening of cognition and gait because of the underlying degenerative disorder’s unrelenting progression [121]. The rate of firing in the globus pallidus increases with DBS of the subthalamic nucleus for Parkinson’s disease; however, excitatory STN neurons that project to the globus pallidus are suppressed [158]. Furthermore, neurons in the globus pallidus display spiking activity that aligns with the stimulus pulse. DBS is hypothesized to suppress dendritic/somatic activity while nearly stimulating axonal output activity [159].

Transcranial magnetic stimulation

Shirota et al. reported an improvement, indicated by a decrease of 6.84 points in the Unified Parkinson’s Disease Rating Scale (UPDRS) Part III, after 1 Hz rTMS of the Supplementary Motor Area 12 weeks post-intervention [122]. The molecular mechanisms behind these effects are not fully understood, despite some theories being suggested. The initial alterations in neuronal ionic conductivity caused by electrolysis events caused by propagating electromagnetic currents appear to be associated with short-term consequences [160]. The release of neurotransmitters is another potential mechanism for short-term effects. High-frequency rTMS administered to the left dorsolateral prefrontal cortex has been linked to tonic dopamine release in the ipsilateral caudate and orbitofrontal cortex [161]. Meanwhile, the long-term effects of TMS are thought to be mediated via neuroplastic processes. The word “neuroplasticity” refers to the CNS’s ability to respond to a wide range of external and internal stimuli via a functional, dynamic remodeling of its structures and connections [155]. This finding is particularly important for PD patients because dopamine availability significantly affects cortical excitability and neuroplasticity. Furthermore, the use of dopaminergic therapy can alter the neurophysiological and behavioral outcomes of stimulation [162].

##### Alzheimer’s Disease

Alzheimer’s disease (AD) affects roughly 3% of the population between the ages of 65 and 74 and more than 50% of the population over the age of 85 [163]. Symptoms usually begin with issues with episodic memory, and as the disease develops, additional cognitive domains, such as language and executive function, are impaired [164]. Pathologically, distinguishing features of AD include the presence of tau neurofibrillary tangles (NFTs) intracellularly (in the neurons) and extracellular amyloid-(A) plaques.

Deep Brain Stimulation

In animals and/or humans, possible targets include the fornix, entorhinal cortex (EC), nucleus basalis of Meynert (NBM), anterior thalamic nuclei, mammillothalamic tract, hippocampus, and ventral capsule [165,166,167]. EC stimulation reduced impairments in a variety of spatial and recognition memory tests in both young and old animals in the TgCRND8 and 3Tg mouse models of AD [123]. Biological findings included increased neurogenesis as well as decreased plaque burden and A peptide concentration, albeit with these effects on amyloid pathology possibly being age dependent [168]. In preliminary trials, the activation of hippocampus outflow channels resulted in significant reversals of hypometabolism and the stability of cognitive deterioration in certain individuals. To date, the majority of publications have been prospective, demonstrating that DBS in memory pathways can have physiological, network-wide metabolic changes and alter various elements of memory function.

Transcranial Magnetic Stimulation

Using rTMS for AD was shown to improve short-term cognitive function (using Alzheimer’s Disease Assessment Scale-Cognitive Subscale and Mini-Mental State Examination) [124,169]. Specifically, compared to other modalities of electrical stimulation, high-frequency rTMS demonstrated better short-term outcomes for general cognitive function. Additionally, the stimulation of multiple sites, rather than single-site stimulation, was reported to have a more pronounced effect on cognition, with a longer duration of treatment of 10 or more sessions and increasing frequency (20 Hz compared to 10 Hz/1 Hz) causing more improvement in the cognitive scores [124].

##### Depression

Major depression is a frequent and difficult disorder that can have a significant impact on quality of life, everyday functioning, and, ultimately, life expectancy [170].

Transcranial direct current stimulation

Transcranial direct current stimulation was studied as a therapy for serious depression, but its efficacy is still debated due to inconsistencies in published data. Several investigations of depression scores after tDCS reported that applying anodal stimulation to the left DLPFC leads to an improvement in the scores that persists for approximately 30 days post-treatment [125], whereas other studies reported no significant impact on cognitive function independent of improvements in mood [171], implying that designing trials that identify the exact impact of tDCS on cognitive function is difficult due to interfering factors like general improvements in cognition during the course of the investigation. Using an individualized approach to optimize DLPFC stimulation, as well as researching the possible value of targeting other key and relevant cortical areas related to emotions and mood through tDCS [172], may increase the efficacy of tDCS for depression therapy.

## 4. Conclusions and Future Trends

The exploration of neuromodulation techniques in the context of the GBA offers promising avenues for addressing a spectrum of neurological and gastrointestinal disorders. The bidirectional communication between the gastrointestinal tract and the CNS, orchestrated by the GBA, underscores the intricate interplay between physiological processes and cognitive functions. Neuromodulation emerges as a versatile and effective therapeutic approach, showing promise in the management of drug-resistant epilepsy, treatment-resistant depression, and FGIDs. The significance of VNS in influencing gastrointestinal motility, permeability, and protection against inflammatory damage signifies its role as a natural treatment for conditions like Crohn’s disease and ulcerative colitis. While challenges and potential complications exist, the potential of neuromodulation to modulate abnormal circuit activity, alleviate chronic abdominal pain, and manage inflammatory processes in various GI disorders highlights its impactful role in improving the quality of life for individuals grappling with these challenging conditions. Looking ahead, the future of neuromodulation in tackling gastrointestinal disorders holds exciting prospects. Ongoing research suggests that the application of neuromagnetic therapies, device-assisted treatments (DATs), and electroacupuncture therapy could further refine our understanding of gut–brain connections and potentially open new avenues for therapeutic interventions. As the field continues to advance, it is anticipated that fine-tuned neuromodulation techniques, such as transcutaneous auricular VNS (tVNS) and tripolar SCS, will play an increasingly pivotal role in reducing inflammation, improving symptoms, and enhancing the quality of life for individuals with FGIDs like IBS. Additionally, the ongoing exploration of DBS and TMS for modulating abnormal circuit activity and addressing chronic pain and inflammatory processes in gastrointestinal disorders suggests continued evolution in the application of neuromodulation techniques for improved patient outcomes.

## Figures and Tables

**Table 1:** Effects of neuromodulation on different diseases associated with GBA changes.

Disease	Study	Type of the Study (Human or Animal)	Reported Effects of Neuromodulation
Irritable bowel syndrome (IBS)	[107]	Human (42 patients)	tVNS increases bowel movements, alleviates abdominal discomfort, enhances IBS symptoms and quality of life, and improves rectal sensation and rectal distention-induced relaxation of the internal anal sphincter.
	[108,109]	Animal	Spinal Cord Stimulation alleviates IBS pain, potentially by suppressing dorsal column pain pathways and selectively suppressing the response to visceronoxious stimulus.
	[110]	Human tissue	Direct electric stimulation activates inhibitory neurons releasing nitric oxide and purine; stimulates excitatory neurons releasing acetylcholine and tachykinins in human intestinal strips.
Inflammatory bowel disease (IBD)	[111]	Animal	VNS reduces inflammation in the gut.
	[112]	Animal	VNS decreases inflammation in the small intestine across normal, spleen-denervated, and T-cell-deficient mice. It mediates effects through targeting resident macrophages.
Gastroesophageal reflux disease (GERD)	[113]	Human	Acute TEA (at bilateral ST36 and PC6 acupoints) enhances stomach accommodation and pace-making activity and reduces post-prandial dyspepsia in GERD.
	[114]	Human	Four-week TEA (at ST36 and PC6) alleviates reflux symptoms, increases distal esophageal motility, enhances stomach accommodation, and reduces inefficient esophageal contractions.
	[115]	Human	Transcutaneous abdominal electrical stimulation reduces acid exposure and DeMeester score by over 50% in PPI-resistant GERD patients.
Autism spectrum disorder (ASD)	[116,117]	Human	VNS in people with autism improves mood after 12 months compared to non-autistic individuals. VNS significantly increases BDNF levels, enhancing neural plasticity in people with autism.
	[118,119,120]	Human	rTMS (of DLPFC) reduces repetitive behaviors and irritability and enhances neurophysiological perception. SMA stimulation improves movement-related potentials. Premotor cortex stimulation enhances sensorimotor integration.
Parkinson’s disease	[121]	Human	STN DBS provides long-term symptom relief in neurodegenerative disorders, despite cognitive and gait decline over time.
	[122]	Human	TMS (for SMA in PD) reduces UPDRS part III scores by 6.84 points at 12 weeks post-intervention.
Alzheimer’s disease	[123]	Animal	Entorhinal cortex stimulation reduces memory impairments in spatial and recognition tasks in young and old animals.
	[124]	Human	TMS for AD improves short-term cognitive function and has enhanced benefits with multi-site, long-term, high-frequency stimulation.
Depression	[125]	Human	Anodic tDCS (of the left DLPFC) may reduce depression scores for up to 30 days.
Schizophrenia	[126]	Human	Anodic (excitatory) stimulation of the left DLPFC in conjunction with cathodic (inhibitory) stimulation of the left temporal–parietal junction was found to significantly reduce auditory verbal hallucinations in schizophrenia.
	[127]	Human	Anodic tDCS to left DLPFC improves probabilistic association learning in schizophrenia.

Transcutaneous auricular vagus nerve stimulation (tVNS), irritable bowel syndrome (IBS), vagus nerve stimulation (VNS), transcutaneous electrical acustimulation (TEA), acupuncture points ST36 (stomach 36) and PC6 (pericardium 6), gastroesophageal reflux disease (GERD), proton pump inhibitor (PPI), brain-derived neurotrophic factor (BDNF), Repetitive Transcranial Magnetic Stimulation (rTMS), dorsolateral prefrontal cortex (DLPFC), supplementary motor area (SMA), Subthalamic Nucleus Deep Brain Stimulation (STN DBS), Parkinson’s disease (PD), Unified Parkinson’s Disease Rating Scale (UPDRS), Alzheimer’s disease (AD), and transcranial direct current stimulation (tDCS).

## Data Availability

No new data were created or analyzed in this study. Data sharing is not applicable to this article.
